# What Doesn’t Kill Them Makes Them Stronger: The Impact of the Resistance Patterns of Urinary *Enterobacterales* Isolates in Patients from a Tertiary Hospital in Eastern Europe

**DOI:** 10.3390/antibiotics11050548

**Published:** 2022-04-20

**Authors:** Ionela-Larisa Miftode, Maria-Antoanela Pasare, Radu-Stefan Miftode, Eduard Nastase, Claudia Elena Plesca, Catalina Lunca, Egidia-Gabriela Miftode, Amalia-Stefana Timpau, Luminita Smaranda Iancu, Olivia Simona Dorneanu

**Affiliations:** 1 Department of Infectious Diseases, Faculty of Medicine, University of Medicine and Pharmacy Gr. T. Popa, 700115 Iasi, Romania; larisa.miftode@yahoo.com (I.-L.M.); edubacau@gmail.com (E.N.); claudia23badarau@yahoo.com (C.E.P.); 2 St. Parascheva Clinical Hospital of Infectious Diseases, 700116 Iasi, Romania; antoanela.pasare@gmail.com (M.-A.P.); odorneanu@yahoo.com (O.S.D.); 3 Department of Internal Medicine I (Cardiology), Faculty of Medicine, University of Medicine and Pharmacy Gr. T. Popa, 700115 Iasi, Romania; darieamalia@gmail.com; 4 Department of Preventive Medicine and Interdisciplinarity, Faculty of Medicine, University of Medicine and Pharmacy Gr. T. Popa, 700115 Iasi, Romania; catalinalunca@yahoo.com (C.L.); luminita.iancu@umfiasi.ro (L.S.I.)

**Keywords:** *Enterobacterale*s, carbapenem-resistance, difficult-to-treat infections, urinary tract infections

## Abstract

(1) Background: The evolution of bacterial resistance to antibiotics is one of the factors that make infectious pathology an extremely dynamic field, also inducing a significant burden on public health systems; therefore, continuous updates on the bacterial resistance to antibiotics and their particular regional patterns is crucial for the adequate approach of various infectious diseases. (2) Methods: We retrospectively analyzed 354 patients with *Enterobacterales* urinary tract infections (UTIs), determined their antibiotic resistance pattern, thus aiming to correlate them with the outcome and other specific markers of poor prognosis. (3) Results: The most frequent causative agent was *Escherichia coli*, representing 64.6% of all UTIs. We identified 154 patients resistant to multiple antibiotic classes, of which 126 were multidrug-resistant (MDR), 17 were extensive drug-resistant (XDR) and 11 were pandrug-resistant (PDR). Moreover, 25 isolates were resistant to carbapenems (CRE), 25 were difficult-to-treat (DTR), and 84 were extended-spectrum cephalosporin-resistant (ESC), with only 95 isolates susceptible to all tested antibiotics. Mortality ranged from 1% for UTIs caused by isolates susceptible to all tested antibiotics, to 24% for the ones caused by DTR or CRE isolates. Other significant risk factors associated with mortality were: prolonged hospital stay (*p* = 0.0001), Charlson comorbidity index ≥ 3 (*p* = 0.02), urinary catheterization (*p* = 0.001), associated respiratory pathologies (*p* = 0.004), obesity (*p* = 0.047), a history of previous hospitalizations (*p* = 0.007), inappropriate empiric antibiotic regimen (*p* = 0.001), or hyper inflammatory status (*p* = 0.006). Basically, we observed that a multiple regression model comprising urinary catheterization, inappropriate empiric anti-biotherapy, obesity, and respiratory comorbidities exhibits the best correlation with mortality rate in patients with UTI (R = 0.347, R^2^ = 0.12). (4) Conclusions: By focusing on the novel resistance patterns, our study provides complementary evidence concerning the resistance profiles found in an Eastern European region, as well as their prognostic implications in patients with UTI.

## 1. Introduction

Urinary tract infections (UTIs) are a common public health issue in both community and nosocomial settings, affecting ~150 million people worldwide each year [1]. UTIs are caused by a wide range of pathogens, both Gram-negative and Gram-positive bacteria, as well as by certain fungi, but usually by uropathogenic *Escherichia coli* [2,3]. UTI is among one of the most common bacterial infections, occurring particularly in women [4].

The development of the antimicrobial agents, which started with the discovery of penicillin by Sir Alexander Fleming in 1928, is a cardinal step in the history of medicine, allowing to prevent millions of deaths due to infectious diseases [5]. Unfortunately, shortly after their discovery, antibiotic resistance emerged, nowadays representing a significant burden to global public health [6].

Integrative approach of UTI has an important role in improving prognosis, implying that in most cases antimicrobial therapy has to be prescribed empirically. In order to provide suitable empirical therapy, it is essential to know the main bacteria typically involved in the urinary tract infection, as well as their antimicrobial resistance pattern. This approach allows limiting antimicrobial resistance and the spread of multidrug-resistant bacterial strains [7].

Numerous international experts came together through a joint initiative by the European Centre for Disease Prevention and Control (ECDC) and the United States Centers for Disease Control and Prevention (CDC), to create a standardized international definition for the description of acquired resistance profiles, as follows:

- MDR (Multidrug Resistance) is defined as acquired non-susceptibility to at least one agent in three or more antimicrobial categories;
- XDR (Extensive Drug Resistance) as non-susceptibility to at least one agent in all but two or fewer antimicrobial categories (i.e., bacterial isolates remain susceptible to only one or two categories);
- PDR (Pandrug-resistance)—non-susceptibility to all agents in all antimicrobial categories [8].

Furthermore, McDonnell et al. [9] added to this list by proposing the notion of UDR: Usual Drug Resistance, to describe isolates that are not fully susceptible wild-type strains but that can nonetheless be readily treated with standard therapies. 

More recently, Kadri et al. [10] proposed the expression of DTR: Difficult to Treat Resistance. Their point of departure is the idea that the MDR-XDR-PDR definitions make no distinction between strengths and weaknesses of the individual antibiotics: agents with higher efficacy and lower toxicity are considered in the same way as agents with lower efficacy and higher toxicity. Therefore, they define DTR as intermediate or resistant to all of the typical first-line, lower toxicity agents, defined as the beta-lactams (including carbapenems and combinations with beta-lactamase inhibitors) and the fluoroquinolones. To determine the DTR status, the susceptibility testing of at least one carbapenem, one extended-spectrum cephalosporin, and one fluoroquinolone is required.

The latest CDC report provided definitions for two other antimicrobial resistance patterns [11]:

- ESC (Extended-spectrum cephalosporin-resistant)—Any *E. coli*, *Klebsiella oxytoca* or *Klebsiella pneumoniae* that has tested Intermediate (I) or Resistant (R) to at least 1 of the following: cefepime, ceftriaxone, cefotaxime, ceftazidime, ceftolozane/tazobactam or ceftazidime/avibactam;
- CRE (Carbapenem-resistant *Enterobacterales*)—Any *E. coli*, *Klebsiella aerogenes, Klebsiella oxytoca, Klebsiella pneumoniae*, or *Enterobacter* spp. that has tested Resistant (R) to at least 1 of the following: imipenem, meropenem, doripenem, ertapenem, meropenem/vaborbactam, or imipenem/relebactam.

The impact of multidrug-resistant Gram-negative bacteria (MDR-GNB) infections can be determined by assessing the clinical outcomes, such as length of stay in the hospital or mortality rates. Although most of the studies found significant correlations between MDR-GNB and mortality risk, other authors failed to demonstrate such an association, therefore the topic remains controversial a fertile ground for further research [12,13,14,15].

This research is a sequel of our previous observations, which focused on the characterization of UTIs caused by *K. pneumoniae*, motivated by the necessity of determining the resistance patterns for all *Enterobacterales* against common antibiotic classes in treating UTIs.

Therefore, the aim of this study was to assess the antibiotic susceptibility rates and epidemiology of UTIs caused by *Enterobacterales*. Moreover, by using the novel resistance patterns, we aim to introduce them in the clinical practice, as a useful and more accurate tool for the characterization of antibiotic resistance in UTIs, especially for the clinicians.

## 2. Materials and Methods

A retrospective cohort study was conducted from 30 June 2019, to 30 December 2019, at “St. Parascheva” Clinical Hospital of Infectious Diseases from Iasi, a 300 beds university setting, as it is the largest tertiary center for Infectious Diseases from North-Eastern Romania, a region with approximately 4 million inhabitants.

### 2.1. Study Population

In this study, we enrolled all hospitalized patients presenting a confirmed UTI, both community-acquired and hospital-acquired (i.e., >48 h after admission), with the following inclusion criteria: (i) suggestive clinical syndrome (dysuria, pollakiuria or non-specific symptoms for catheterized patients, such as fever or chills); (ii) pyuria [≥10 white blood cell count (WBC)/mm^3^]; (iii) isolation of GNB *Enterobacterales*, including *E. coli*, *K. pneumoniae,* and *Enterobacter* spp., *Proteus* spp., *Serratia* spp*., Providencia* spp*. or Morganella* spp. in urine culture [≥10^5^ colony forming units (CFU)/mL)]. We included only one isolate per patient (except for the isolates with different antibiotic susceptibility, which were considered as different isolates) and excluded those with GNB *Enterobacterales* colonization or with a urinary CFU count <10^5^/mL. 

A total of 354 clinical specimens were analyzed during the study period. Bacterial identification was automated, using phenotypical characters. Based on the antibiotic resistance pattern, we classified the isolates as susceptible to all tested antibiotics (S), MDR, XDR, and PDR. We also further divided the strains according to the novel resistance pattern such as UDR, DTR, CRE, or ESC.

### 2.2. Data Collection

Patient information was collected from the medical records, including age, gender, type of bacteria (*E. coli*, *K. pneumoniae, Enterobacter* spp. *Proteus* spp., *Serratia* spp*., Providencia* spp., or *Morganella* spp.*)*, resistance pattern for each isolate, previous hospitalizations (within the past 3 months) or use of antibiotics within the past 30 days, urinary catheterization, presence of comorbid conditions (such as kidney disease, or diabetes mellitus), treatment regimen, and clinical outcome. 

### 2.3. Microbiological Procedures

Antimicrobial susceptibility testing was performed by the Kirby Bauer disk diffusion method, using the following antimicrobial discs: ampicillin, amoxicillin/clavulanic acid, piperacillin/tazobactam, cefixime, cefuroxime, ceftazidime, ceftriaxone, cefotaxime, cefoxitine, cefepime, imipenem, meropenem, ertapenem, amikacin, gentamicin, tobramycin, ciprofloxacin, norfloxacin, moxifloxacin, ofloxacin, trimethoprim/sulfamethoxazole, and nitrofurantoin. We used EUCAST clinical breakpoint table v9.0 for the interpretation of the minimal inhibitory concentrations (MIC) and zone diameters.

### 2.4. Carbapenemase Detection

For isolates resistant to carbapenems, NG-Test Carba 5 multiplex lateral flow immunoassay was used for the phenotypic detection and differentiation of five common carbapenemase families: *Klebsiella pneumoniae* carbapenemase (KPC), oxacillinase (OXA-48-like), Verona integron encoded metallo-β-lactamase (VIM), imipenemase (IMP), and New Delhi metallo-β-lactamase (NDM). The test was performed according to the manufacturer’s instructions.

### 2.5. Statistical Analysis 

We used Kolmogorov–Smirnov test to assess the normal distribution of parameters in the study population, normally distributed variables being presented as means ± standard deviation. Categorical variables are presented as absolute numbers or percentages. The differences between various subgroups were assessed using independent *t*-test or one-way ANOVA, as appropriate. For certain significant differences objectified within subgroups following the ANOVA analysis, we performed a post hoc Dunnett’s test. The correlation analysis between two or more variables was performed using either Pearson’s (for continuous variables) or Spearman’s (for categorical variables) rank (r) coefficients. 

To compare the survival distribution within the resistance pattern subgroups we used the log-rank test, while Kaplan–Meier method was used for the estimation of the survival curves.

A multivariate logistic regression was also performed to identify a specific model comprising multiple risk factors as predictors associated with multidrug resistance (dependent variable), with Hosmer–Lemeshow goodness-of-fit test indicating that the model adequately describes the analyzed data. 

A *p*-value of 0.05 was considered statistically significant. For the initial data collection, we used Microsoft Excel 2013 version (Microsoft Corporation, Redmond, WA, USA), while the data analysis was performed with SPSS version 23 (IBM, Armonk, VA, USA).

## 3. Results

### 3.1. Etiology of UTIs and the Pathogen’s Resistance Profile

The 354 specimens were analyzed during the study period. The most frequent causative agent was *E. coli*, representing 64.6% of all cases (229 strains), followed by *Klebsiella*, encountered in 21.4% cases (76 strains; 72—*K. pneumoniae* and 4—*K. oxytoca*), *Proteus* spp.*—*9.6% (34 cases; 32—*Proteus mirabilis* and 2 *Proteus vulgaris*), and *Enterobacter* spp.—2.8% (10 cases; 8—*Enterobacter* spp. and 2—*Enterobacter cloacae*); we also identified *Providencia* spp. and *Serratia marcescens*, in 2 cases each and *Morganella morganii* in only one case.

We identified a worrisome share of 43.5% isolates resistant to multiple antibiotic classes (154 patients), of which 126 were MDR, 17 were XDR, and 11 were PDR. Moreover, 25 isolates were CRE and 84 were ESC, with only 95 isolates susceptible to all antibiotics tested. Of the 25 DTR strains, 2 (8%) were MDR, 12 (48%) were XDR, and 11 (44%) were PDR, while in the UDR group, there were only 20 (16.5%) MDR strains without any PDR or XDR. In the CRE group, we found 6 (24%) MDR, 12 (48%) XDR, and 7 (28%) PDR strains, and from the ESC group, 62 (73.8%) strains were MDR, 14 (16.6%) were XDR, and 7 (8.3%) were PDR (Table 1).

Patients with UTIs due to resistant strains were more likely men, aged over 65 years, with recent hospitalizations (within the past three months) and previous antibiotic intake (within the last month). In addition, they were more likely to have an indwelling urinary catheter, an increased inflammatory status (expressed as a high C-reactive protein-CRP), with a significantly longer length of hospital stay. In addition, we observed a gradient of the mean values of Charlson comorbidity index (CCI) following the susceptibility patterns’ spectrum; the overall mean value was 3.4, the lowest (2.6) was encountered in the S group, while the highest values were observed in PDR group (5.6), CRE, DTR (5 each), and XDR (4.9), respectively. In this regard, as components of CCI, we noted that chronic kidney disease (CKD) was more prevalent among patients with resistant strains, while diabetes mellitus (DM) was affecting a roughly similar share of patients, irrespective of their resistance profile (Table 2). 

### 3.2. Particular Aspects of the Antibiotic Resistance and Antibiotherapy

*Enterobacterales* species showed different resistance patterns to certain antibiotics, as presented in Table 3. *Except* for *E. coli*, all isolates showed low susceptibility rate (below 70% or 80%) to all beta-lactams, the carbapenems being the only notable exception. A severe susceptibility pattern was found in *Klebsiella* isolates, with significant resistance to beta-lactams (except for imipenem and meropenem), fluoroquinolones, and all aminoglycosides.

Out of 354 isolates, 25 (7%) were CRE, with 19 isolates being carbapenemase producers, as determined by the NG-Test Carba 5 multiplex immunoassay. Of these, 17 isolates were *K. pneumoniae*, one was *E. coli* (NDM), and one was *Enterobacter cloacae* (OXA-48). The most commonly identified carbapenemase was OXA-48 (8 isolates), followed by NDM (7 isolates), KPC (3 isolates), and VIM (1 isolate).

**Figure 1:**
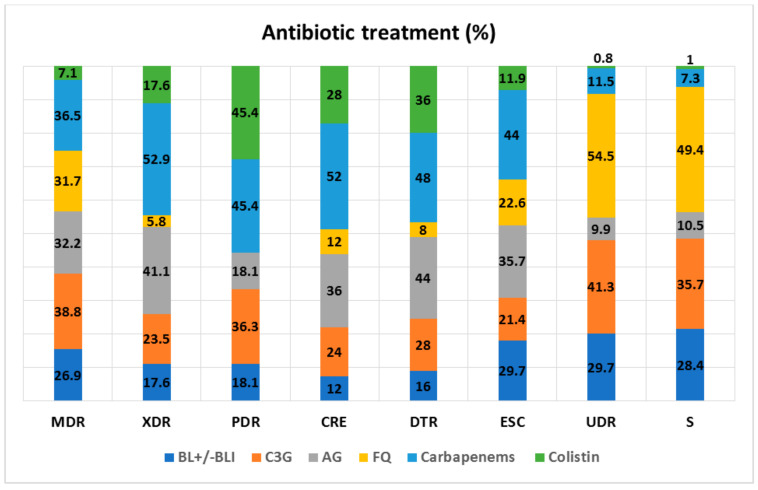
Antibiotic treatment. BL+/−BLI—Beta-lactam+/−Beta-lactamase inhibitor; C3G—3rd generation cephalosporin; AG—aminoglycosides; FQ—fluoroquinolones; MDR—Multi-drug Resistant, XDR—Extensive Drug Resistant, PDR—Pandrug-resistant, UDR—Usual Drug Resistance, DTR—Difficult to Treat Resistance, S—Susceptible to all the tested antibiotics, ESC—Extended-spectrum cephalosporin-resistant, CRE—Carbapenem-resistant *Enterobacterales*.

Given the high prevalence of resistant strains, the most commonly used antibiotic classes were carbapenems, followed by third generation cephalosporins and aminoglycosides. Beta-lactams (+/−beta-lactamase inhibitors) and fluoroquinolones were used mostly for UDR and S infections, while colistin was predominantly administered in patient with PDR or DTR (Figure 1). 

### 3.3. Resistance Profile and the Prognosis Assessment

Mortality rate amongst patients with resistant strains was significantly higher compared to their S counterparts (1.05% vs. 8.9%, *p* = 0.009) (Table 4). 

**Figure 2:**
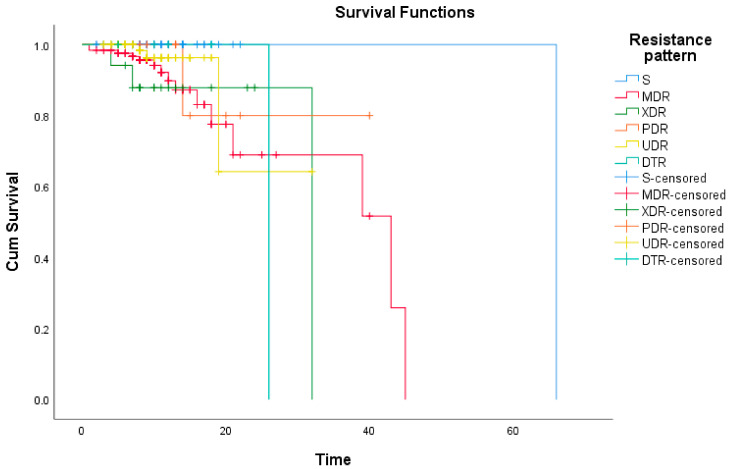
Kaplan–Meier survival curves for specified resistance patterns; MDR—Multi-drug Resistant, XDR—Extensive Drug Resistant, PDR—Pandrug-resistant, UDR—Usual Drug Resistance, S—Susceptible to all the tested antibiotics.

Log-rank test revealed the increased risk of death amongst patients with resistant strains compared to S ones (*p* = 0.002). The subsequent detailed Kaplan–Meier survival curves confirmed the high mortality risk associated with resistant strains, but failed to draw a specific prognosis pattern concerning different types of antibiotic resistance (Figure 2).

Consequently, an additional ANOVA test revealed a significant difference concerning mortality rate within resistance groups. By performing a detailed Dunnett post hoc analysis, we observed that MDR and XDR profiles were associated with a significant excess of mortality compared to susceptible strains. Although PDR and UDR also showed higher mortality rates, they did not reach the threshold of statistical significance, while DTR group was slightly above it (Table 5).

Concerning the recently introduced CDC resistance patterns, we found that both CRE and ESC were positively and significantly correlated with mortality, but also with other markers of severity, such as length of stay, indwelling urinary catheter and Charlson comorbidity index. Moreover, the two resistance patterns were significantly correlated with each other (r= 0.387, *p* < 0.001). The same trend was also observed for DTR, which presented significant positive correlations with the above-mentioned factors of poor outcome (Table 6).

However, we did not identify any notable differences between the types of carbapenemases concerning the vital prognosis or the presence of other markers of severity. Neither OXA, NDM, KPC, or VIM were not significantly associated with an excess of mortality (*p* > 0.05 for all paired comparisons).

### 3.4. Additional Poor Prognosis Factors in UTIs

Further, we aimed to analyze the correlation between mortality rate and certain risk factors commonly associated with UTIs. We found that comorbidities play a major role in the outcome of these patients, as Charlson comorbidity index, respiratory pathologies and obesity presented significant direct correlations with the mortality risk (Table 7). On the other hand, female sex was significantly associated with an improved survival rate (r = −0.106, *p* = 0.045). Very importantly, a history of previous hospitalizations or urinary catheterization, as well as an inappropriate empiric antibiotic regimen or hyper inflammatory status (expressed as high levels of C-reactive protein) also exhibited strong correlations with mortality rates in our study group. On the other hand, some traditionally incriminated UTI risk factors, such as smoking, diabetes mellitus, or cardiovascular diseases were not significantly correlated with a poor outcome.

Given that all these aspects associated with a poor prognosis may coexist in varying proportions in the same patient, through a multiple regression we aimed to create a model for more accurate prediction mortality. We observed that a model comprising urinary catheterization, inappropriate empiric antibiotics, obesity, and respiratory comorbidities has the best correlation with mortality (R = 0.347). Basically, the R^2^ of 0.12 express that 12% of mortality variance may be explained by this composed model. Very interestingly, when included in multiple regression, CCI was no longer a significant predictor of poor outcome and, therefore, cannot be added in the above-mentioned model (Table 8).

## 4. Discussions

This study focused on the widespread prevalence of various drug-resistance patterns among *Enterobacterales* UTIs in North-Eastern Romania and their impact on the patients’ outcome. This is one of the first studies that specifically addressed DTR as a novel resistance pattern in UTIs, the other currently available data referring mainly to bloodstream infections.

As expected, the most commonly identified uropathogen was *E. coli*, responsible for almost two-thirds of the recorded cases of UTI. This finding is in accordance with multiple recent studies [16,17,18,19], which found it to be the main etiological agent in up to 95% of the UTIs. The second most isolated bacteria were *Klebsiella* spp., encountered in 76 cases (21.4%), of which *K. pneumoniae* was by far the most common species, an aspect in accordance with the results reported by several recent studies [4,17,18,20], even though a recent study conducted in another region of Romania reported *K. pneumoniae* as the leading etiology in some settings [21].

Regarding the antibiotic resistance pattern, we identified that 43.5% of isolates were resistant to multiple antibiotic classes. Of those, the majority was MDR (35.5%), followed by XDR (4.8%) and PDR (3.1%). The identification of PDR strains represents a major concern; multiple studies that analyzed the resistance profile of Gram-negative pathogens have failed to identify such a pattern [13,22], while others have reported negligible amounts [23,24].

Another goal of the study was to turn the spotlight on the novel resistance classification, aiming to outline a local epidemiological profile, since Romania constantly top ranks in terms of antibiotic resistance [25]. The DTR pattern can be described as a subcategory within MDR/XDR/PDR strains, being resistant to all first-line agents: entire range of β-lactams (including carbapenems), various combinations with β-lactamase inhibitors, as well as to fluoroquinolones. A plethora of studies has tried so far to assess the prognostic utility of this novel resistance pattern, but were focusing mainly on blood stream infections. Benkő et al. analyzed the prevalence of DTR strains among the ESKAPE pathogens and found only 23 isolates (0.46%), mostly *Acinetobacter baumannii* and *Pseudomonas aeruginosa*, with only one *Klebsiella* spp. and one *Proteus* spp. [26]. Similarly, Gianella et al. conducted a research in the region of Emilia-Romagna, Italy, and identified higher numbers of DTR patterns, representing 11% of all isolates, with *K. pneumoniae* accounting for all but one strains [23], while reports from France [27], Hungary [28] or United States [10] highlighted a much lower prevalence of DTR, constantly reported as maximum 1%. Interestingly, there is a wide spectrum of DTR variance among bacterial species, the highest shares of DTR being found among UTIs caused by *A. baumannii* [10]. In our study, we found that out of the 25 DTR strains (7% of all cases, of which two were MDR, 12 were XDR, and 11 were PDR), most of the isolates were *K. pneumoniae*, followed by *Enterobacter* spp., *Providencia* spp., *Proteus* spp. and *Serratia* spp. 

The already established risk factors for the acquisition of an UTI caused by a drug-resistant pathogen, as reported in numerous studies conducted worldwide [29,30,31] are age, male gender, previous hospitalizations, previous antibiotic use, and urinary catheterization, all of them being also significantly higher among patients with resistant isolates from our study, especially in the PDR and DTR groups. Furthermore, Faine et al. identified some other risk factors, such as chronic hemodialysis and nursing home residence [32], while Ben Ayed et al. found a correlation between the presence of diabetes mellitus or a history of urinary tract surgery in the last 12 months, with the acquisition of a MDR community-acquired UTI [33]. 

Moreover, Tenney et al. [34] performed a systematic review including more than 30.000 patients, aiming to stratify the risk factors for MDR UTIs, according to their prevalence; in this regard, they classified the risk factors as probable (urinary catheterization, previous hospitalization, or antibiotic intake and nursing home residence), possible (age, history of UTI and male gender) and unlikely/supplementary research needed (diabetes mellitus, recent travel, ethnicity, immunosuppression, and female gender). This classification is consistent with our findings; all the noteworthy risk factors we identified fit in the “probable” and “possible” categories, while those categorized as “unlikely” did not reach the statistical significance; worth mentioning, female gender was a protective factor, being strongly associated with S isolates and, consequently, with a favorable outcome.

When assessing individual antibiotic susceptibility, we identified a great rate of isolates resistant to all aminopenicillins (+/−IBL) and fluoroquinolones, given the fact that these antibiotics usually represent the empiric treatment for UTIs in the north east region of Romania. We also identified a high resistance rate to aminoglycosides, particularly to gentamicin and tobramycin, while amikacin remains active for most isolates of *E. coli* and *Proteus* spp. Carbapenems continue to be a viable option for most Gram-negative UTIs, even though we identified a total of 25 strains, mostly *K. pneumoniae*, resistant to carbapenems (of which 19 were carbapenemase-producers).

Our results are more dramatic than those reported in most of the studies [35,36,37,38], even though we have found some others with higher resistance rates [19,24,39]. For example, Kot et al. identified all *E. coli* and *P. mirabilis* isolates as susceptible to carbapenems (vs. 99.1% and 91.1%, respectively, in our study), and an 84.4% susceptibility for *K. pneumoniae* isolates (vs. 68.4% sensitivity to ertapenem in ours) [17]. Even though *Proteus* spp. is usually associated with hospital-acquired UTIs [21], with higher resistance rates to carbapenems [40,41], we identified up to 97% susceptibility to meropenem, similar to other Romanian research [16]; however, we must interpret these results with caution, due to the relatively small number of *Proteus* spp. isolates. Interestingly, a recent study that included isolates from other regions of Romania, reported a significantly lower resistance rates for both *E. coli* and *K. pneumoniae* UTIs in females, with 85.3% of *E. coli* and 73.9% of *K. pneumoniae* isolates being susceptible to amoxicillin-clavulanic acid (vs. 65% and 32.8%, respectively, in our study), 91.2% and 81.8% presenting sensitivity to ceftazidime (vs. 80.7% and 48.6% in ours), 96% and 88.4% to amikacin (vs. 71.1% and 44.7%) and 98.16% and 93.9% sensitivity to Imipenem (vs. 100% and 82.8%) [42]. However, Sokhn et al., while investigating the resistance pattern of Gram-negative uropathogens in Lebanon, found similar or even lower sensitivity to some of the antibiotics tested; they reported only 42.4% and 35.8% sensitivity to amoxicillin-clavulanic acid for *E. coli* and *K. pneumoniae*, respectively, 69.4% and 65.7% to ciprofloxacin (vs. 71.1% and 44.7% in our study), 77.6% and 77.8% to amikacin, while 100% of their isolates were susceptible to all tested carbapenems (imipenem and meropenem) [19].

Those regional differences in terms of antibiotic resistance can be based also on the socioeconomic status of the countries where the studies were conducted. Although the resistance mechanisms are triggered by certain microbiological and molecular particularities, those phenomena may be enhanced by specific socioeconomic and behavioral factors. There is a growing body of evidence that antibiotic resistance is positively correlated with a poor economic status, with higher rates of resistance found in less developed countries [43,44,45]. Some socio-governmental determinants that may induce the selection of resistant strain are the limited acknowledge of the standardized protocols concerning the antibiotic prescription by the medical staff, the non-judicious use of broad-spectrum antibiotics in several medical departments, or even the abusive prescriptions by general practitioner irrespective of the involved germ or local epidemiology recommendations. Of course, at individual level, a poor educational status can be the source for frequent, empirical antibiotic self-administration or, conversely, for incomplete adherence to a prescribed antibiotic regimen, resulting in an increased antibiotic resistance [46], as confirmed by the results of our study. Last but not least, an impaired socioeconomic status can be the trigger for various comorbidities that can alter the natural immunity against infections or can even be associated with some precarious housing issues vastly incriminated in the apparition of UTIs (e.g., lack of running water or indoor sanitation facilities) [47]. The region of Romania where this study was conducted can be considered a borderline region, exhibiting characteristics from both the developed and the developing countries, therefore the reported resistance profiles from this area could emerge as relevant epidemiological benchmarks.

The prognosis of patients with UTIs may be significantly influenced by the resistance pattern. Therefore, we noted that the overall in-hospital mortality in our study was 6.7%, ranging from 1% in the S group to 24% in CRE and DTR groups. Risk factors strongly associated with a negative outcome were urinary catheterization, previous recent antibiotic treatment or hospitalizations within the past 3 months, respiratory comorbidities, obesity, longer hospital stay, and inappropriate empirical therapy. Clearly, multiple associated pathologies, as well as older age, all summed in the CCI, have a negative impact on the survival rate. A CCI value ≥3 was positively correlated with a higher mortality risk in our study, a similar cut-off value being reported also by Hoxha et al. [48], while Hussein et al. [49] claimed that a value ≥5 is more appropriate in the mortality risk assessment in patients with carbapenem-resistant *K. pneumoniae* infections. We also observed that a high inflammatory state was correlated with increased risk of death. Even if this biomarker is not specific for UTIs, the baseline and dynamics of CRP’s serum levels represent an adequate tool for risk stratification and prognosis assessment of every admitted patient, regardless the etiology of infection or the resistance pattern [50].

## 5. Conclusions

Our study provides complementary evidence concerning DTR’s regional epidemiological pattern, as Romania and, in general, the whole of Eastern Europe present significant bacterial resistance to various antibiotic classes. The high prevalence of resistant strains and the extensive use of broad-spectrum antibiotics highlighted in our study define not only the alarmingly increasing severity of UTIs in this area, but also the need for prompt strategies concerning their prophylaxis and therapeutic approach. We noted that novel resistance patterns such as DTR, ESC, and CRE are both significantly correlated with a poor outcome in patients with UTI. Therefore, the knowledge of these resistance patterns may represent a cornerstone for a more appropriate antibiotic selection or initial empiric therapy, with a subsequent positive impact on patients’ prognosis and the healthcare-associated burden.

## 6. Limitations

The main limitations of the study were its unicentric design and the relatively small number of cases, due to the fact that, starting with January 2020, the hospital was designated as a COVID-19 support facility, hence the addressability of patients with UTIs significantly decreased.

## Figures and Tables

**Table 1:** Distribution of the causative microorganisms isolated in urine culture by resistance profile in the study population.

Bacterial Species	MDR	XDR	PDR	UDR	DTR	S	CRE	ESC
	126	17	11	121	25	95	25	84
	(35.5%)	(4.8%)	(3.1%)	(34.1%)	(7%)	(26.8%)	(7%)	(23.7%)
*E. coli*	76 (60.3%)	3 (17.6%)	0	92 (76%)	0	74 (77.9%)	2 (8%)	44 (52.4%)
*Klebsiella* spp.	23 (18.2%)	11 (64.7%)	7 (63.6%)	20 (16.5%)	18 (72%)	14 (14.7%)	20 (80%)	40 (47.6%)
*Proteus* spp.	20 (15.8%)	0	1 (9%)	7 (5.7%)	1 (4%)	6 (6.3%)	N/A	N/A
*Enterobacter* spp.	5 (3.9%)	3 (17.6%)	0	2 (1.6%)	3 (12%)	1 (1%)	3 (12%)	N/A
*Serratia* spp.	1 (0.7%)	0	1 (9%)	0	1 (4%)	0	N/A	N/A
*Providencia* spp.	0	0	2 (18%)	0	2 (8%)	0	N/A	N/A
*Morganella* spp.	1 (0.7%)	0	0	0	0	0	N/A	N/A

MDR—Multi-drug Resistant, XDR—Extensive Drug Resistant, PDR—Pandrug-resistant, UDR—Usual Drug Resistance, DTR—Difficult to Treat Resistance, S—Susceptible to all the tested antibiotics, ESC—Extended-spectrum cephalosporin-resistant), CRE—Carbapenem-resistant *Enterobacterales*. N/A—Not Applicable.

**Table 2:** Baseline characteristics of the study population according to the resistance profile.

Parameter	MDR	XDR	PDR	UDR	DTR	S	CRE	ESC
	N = 126	N = 17	N = 11	N = 121	N = 25	N = 95	N = 25	N = 84
Female gender	69 (54.7%)	8 (47%)	5 (45.4%)	93 (76.8%)	10 (40%)	81 (85.2%)	9 (36%)	45 (53.5%)
Previous hospitalizations	41 (32.5%)	10 (58.8%)	8 (71.7%)	20 (16.5%)	16 (64%)	8 (8.4%)	14 (56%)	33 (39.2%)
Previous antibiotic use	19 (15%)	7 (41.1%)	3 (27.2%)	14 (11.5%)	8 (32%)	12 (12.6%)	8 (32%)	19 (22.6%)
Mean age (years)	65.1 ± 15.2	67.3 ± 17.3	74.3 ± 19.2	60.1 ± 21.8	71.4 ± 9.9	57.4 ± 20.5	71.4 ± 10.9	65.5 ± 16.4
Length of stay (days)	11.2 ± 7.2	12.6 ± 7.5	15.6 ± 9.4	9.1 ± 5.0	14.8 ± 8.7	9.2 ± 7.2	14.2 ± 8.8	11.4 ± 7.1
CRP (mg/dL)	9.1 ± 9.7	6.9 ± 7.8	9.4 ± 8.2	8.1 ± 9.5	8.8 ± 7.8	7.6 ± 9.5	6.6 ± 7.3	8.4 ± 8.9
Urinary catheterization	38 (30.1%)	8 (47%)	9 (81.8%)	14 (11.5%)	18 (72%)	10 (10.5%)	14 (56%)	31 (36.9%)
Appropriate empirical antibiotic therapy	51 (40.4%)	6 (35.2%)	5 (45.4%)	78 (64.4%)	9 (36%)	76 (80%)	6 (24%)	32 (38%)
Diabetes mellitus	32 (26.2%)	4 (23.5%)	3 (27.2%)	32 (26.4%)	7 (28%)	20 (21%)	6 (24%)	23 (27.3%)
Charlson comorbidity index	3.8 ± 2.3	4.9 ± 2.6	5.6 ± 2.4	3 ± 2.3	5 ± 2.3	2.6 ± 2.2	5 ± 2.5	4.1 ± 2.6
Chronic kidney disease	17 (13.5%)	7 (41.1%)	2 (18.1%)	8 (6.6%)	7 (28%)	8 (8.4%)	6 (24%)	15 (17.8%)
rUTIs	22 (17.4%)	5 (29.4%)	0	15 (12.3%)	3 (12%)	10 (10.5%)	3 (12%)	16 (19%)
ESBL production	75 (59.5%)	13 (76.4%)	8 (72.7%)	5 (4.1%)	17 (68%)	0	6 (24%)	71 (84.5%)
Mortality	16 (12.6%)	3 (17.6%)	2 (18.1%)	5 (4.1%)	6 (24%)	1 (1%)	6 (24%)	12 (14.2%)

MDR—Multi-drug Resistant, XDR—Extensive Drug Resistant, PDR—Pandrug-resistant, UDR—Usual Drug Resistance, DTR—Difficult to Treat Resistance, S—Susceptible to all the tested antibiotics, ESC (Extended-spectrum cephalosporin-resistant), CRE—(Carbapenem-resistant *Enterobacterales*), CRP—C-reactive protein. rUTIs—Recurrent urinary tract infections, ESBL—Extended spectrum β-lactamases.

**Table 3:** Rates of susceptibility (%) for the most common pathogens isolated.

Type of Bacteria	Tested Antibiotic																
	AMP	AMC	SAM	TMT-SMX	CXM	CAZ	CTX	FEP	GM	TOB	AK	CIP	TZP	IMI	MEM	ETP	COL
*E. coli*	40.1	65.0	71.1	62.8	80.3	80.7	81.2	80.7	86.9	84.2	94.7	71.1	89.9	100	100	99.1	99.5
*Klebsiella* spp.	IR	32.8	36.8	60.5	47.3	48.6	52.6	52.6	56.5	50.0	78.9	44.7	47.3	82.8	82.8	68.4	85.5
*Proteus* spp.	29.4	55.8	52.9	29.4	50	52.9	55.8	73.5	64.7	44.1	94.1	32.5	88.2	91.1	97	94.1	IR
*Enterobacter*	IR	IR	IR	30	20	20	20	20	30	30	80	30	50	80	80	50	90

AMP = ampicillin; AMC = co-amoxiclav; SAM = ampicillin + sulbactam; TMT-SMX = sulfamethoxazole-trimethoprim; CXM = cefuroxime; CAZ = ceftazidime; CTX = cefotaxime; FEP = cefepime; GM = gentamicin; TOB = tobramycin; AK = amikacin; CIP = ciprofloxacin; TZP = piperacillin-tazobactam; IMI = imipenem; MEM = meropenem; ETP = ertapenem; COL = colistin; IR = intrinsic resistance. ***Green***: ≥90% susceptibility rates; these antibiotics can be prescribed empirically even in severe infections, ***Yellow***: susceptibility rate ≥80% but <90%; these antibiotics may be prescribed empirically in mild to moderate infections; ***Red***: susceptibility <80%; these antibiotics should not be prescribed empirically in any kind of infection; ***Grey***: Not applicable

**Table 4:** Mortality rates according to the resistance profile.

	Resistance Profile	N	Fatalities	*p*
Mortality rate	S	95	1 (1.05%)	0.009
	R	259	23 (8.9%)	

**Table 5:** Influence on the mortality rate of various resistance patterns, compared to S strains.

ANOVA (Dunnett 2-Sided)		Mean Difference in Mortality	*p*
MDR	S	0.109	0.006
XDR	S	0.166	0.05
PDR	S	0.080	0.817
UDR	S	0.019	0.895
DTR	S	0.198	0.065

Abbreviations: MDR—Multi-drug Resistant, XDR—Extensive Drug Resistant, PDR—Pandrug-resistant, UDR—Usual Drug Resistance, DTR—Difficult to Treat Resistance, S—Susceptible to all the tested antibiotics.

**Table 6:** Correlations between novel resistance patterns and mortality rates or other markers of severity.

Variable	CRE		ESC		DTR	
	R	*p*	R	*p*	R	*p*
Mortality rate	0.145	0.006	0.138	0.009	0.156	0.003
Length of stay	0.142	0.007	0.122	0.02	0.176	0.001
Urinary catheterization	0.229	0.001	0.201	0.001	0.326	0.0001
Charlson comorbidity index	0.187	0.001	0.158	0.003	0.185	0.001

Abbreviations: DTR—Difficult to Treat Resistance, ESC—Extended-spectrum cephalosporin-resistant, CRE—Carbapenem-resistant *Enterobacterales*.

**Table 7:** Correlations between mortality and specific risk factors for UTI.

Variable	Deceased	
	R	*p*
Age >65 years	0.085	0.109
Length of hospital stay (days)	0.311	0.0001
Charlson comorbidity index ≥ 3	0.164	0.02
Urinary catheterization	0.266	0.001
Female sex	−0.106	0.045
C-reactive protein	0.148	0.006
Rural area	−0.071	0.186
Pregnancy	−0.044	0.416
Smoking	0.020	0.710
Alcohol abuse	0.013	0.810
Respiratory comorbidities	0.154	0.004
Cardiovascular comorbidities	0.034	0.522
Diabetes mellitus	−0.05	0.929
Obesity	0.105	0.047
Previous hospitalizations	0.142	0.007
Previous antibiotic therapy	0.101	0.051
Inappropriate antibiotic empiric therapy	0.193	0.001

**Table 8:** Multiple regression model for predicting in-hospital mortality.

Model Summary ^e^					
Model	R	R Square	Adjusted R Square	Std. Error of the Estimate	*p*
1	0.277 ^a^	0.077	0.074	0.238	0.001
2	0.309 ^b^	0.095	0.090	0.236	0.001
3	0.331 ^c^	0.109	0.102	0.235	0.001
4	0.347 ^d^	0.120	0.110	0.234	0.001

^a^. Predictors: (Constant), Urinary catheterization. ^b^. Predictors: (Constant), Urinary catheterization, Inappropriate antibiotic. ^c^. Predictors: (Constant), Urinary catheterization, Inappropriate antibiotic, obesity. ^d.^ Predictors: (Constant), Urinary catheterization, Inappropriate antibiotic, obesity, respiratory comorbidities. ^e.^ Dependent Variable: Mortality.

## Data Availability

The data presented in this study are available in this article.
